# CARMIL membrane-binding domain regulates capping protein and actin assembly

**DOI:** 10.1016/j.jbc.2026.111484

**Published:** 2026-04-22

**Authors:** Olivia L. Mooren, Patrick McConnell, James D. DeBrecht, John A. Cooper

**Affiliations:** Department of Biochemistry and Molecular Biophysics, Washington University School of Medicine, St Louis, Missouri

**Keywords:** CARMIL, capping protein, membrane binding, basic, hydrophobic motif, actin assembly, Arp2/3 complex

## Abstract

Actin assembly at membranes is associated with protein domains that bind and regulate heterodimeric actin capping protein (CP). CP-binding domains can target CP to the membrane and activate CP by promoting dissociation of its stoichiometric inhibitor V-1. The CP-binding region of CARMIL includes a CPI motif and a CSI motif, followed by a membrane binding (MB) domain. The MB domain is necessary for CARMIL function in cells, and it can target GFP to the plasma membrane. Here, we investigated the mechanism and significance of the relationship of the MB domain to CP activity, including capping of actin filament barbed ends and promotion of Arp2/3-nucleated actin assembly. We found that the MB domain is able to bind to lipid-coated beads, bring the CPI and CSI motifs to the bead, and thus activate CP to promote Arp2/3-based actin assembly. In addition, we discovered that the MB domain can dissociate from the lipid membrane once CP binds; this observation may help account for the long-standing quandary as to how activated CP is released from the membrane and how CP functions to activate Arp2/3-mediated actin assembly near the membrane. Thus, the CARMIL MB domain has multiple biochemical functions regulating actin assembly at a membrane. First, it can target CARMIL, CP, and barbed ends to the plasma membrane. Second, the MB domain can leave the membrane, and this promotes the uncapping of capped barbed ends and activates soluble CP, with greater ability than seen with the membrane-attached state.

Actin filament assembly is necessary for cells to change their shape, move components about the cytoplasm, and migrate from one location to another (1). Assemblies of actin filaments control the location and timing of myosin-based movements, such as contractions of muscle tissue and platelets. In addition, the polymerization of actin filaments from subunits generates forces that drive the movement of membranes, including plasma membranes. These forces change the shape of cells and power their migration.

Regulation of actin filament assembly is provided by proteins that bind directly to actin and by regulatory molecules that bind to actin-binding proteins (2). Assembly of filaments from subunits often occurs at barbed ends (3), which can be bound and controlled by heterodimeric capping protein (CP) (4). Cells regulate the barbed-end capping activity of CP, and this allows them to control actin polymerization and actin-based motility. CP is one of the several components essential for actin assembly mediated by the activation of Arp2/3 complex (5). In addition, CP regulates free barbed ends created by other mechanisms, including nucleation of actin polymerization by formins, EnaVASP, and SPIN90 proteins (6, 7, 8).

The actin capping activity of CP can be regulated in several ways (recently reviewed in (9)). First, CP is inhibited by the small protein V-1 (aka myotrophin), which binds directly to CP on the top of its mushroom-shaped structure. The V-1 binding site overlaps with the binding site for the barbed end of the actin filament, thus sterically blocking interaction with actin. In addition, CP is regulated by a set of proteins with motifs that bind directly to CP at a separate site. These motifs, termed “CP-Interacting” (CPI) motifs (10), are found in a wide variety of otherwise unrelated proteins, many of which localize near membranes (11).

CARMIL proteins are one family of regulators with CPI motifs (11, 12). CARMIL proteins are large single polypeptides with multiple domains. Vertebrate genomes have three highly conserved genes encoding CARMILs. CARMIL protein domains, from N to C terminus, include a noncanonical PH domain, a leucine-rich repeat region, a helical homodimerization domain, a CP-binding region (CBR), and a proline-rich domain (13, 14). The CBR contains a CPI motif, a second CP-binding motif termed the “CARMIL-Specific Interacting” (CSI) motif (10), and a membrane binding (MB) domain with a basic hydrophobic (BH) motif. The three motifs are found in tandem in the unstructured C-terminal half of all vertebrate CARMILs (diagrammed in Fig. 1*A*). CARMILs of invertebrates and protozoa contain the CPI motif but lack the CSI motif and the MB domain (12).

**Figure 1:**
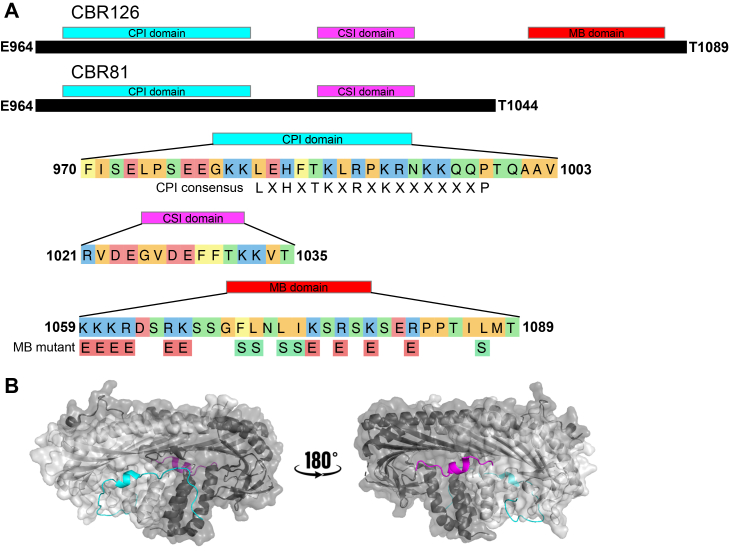
**Domain architecture of the CP-binding region (CBR) of human CARMIL1.***A*, the polypeptides in this study are shown, with the three important regions expanded for additional detail. The conserved CPI consensus sequence is listed below the CPI domain. The set of amino acid mutations used to abrogate membrane binding is shown below the MB domain. *B*, crystal structures (modified from PDB 3LK3) of CP (alpha subunit in *dark gray* and beta subunit in *light gray*) with the CARMIL1 CPI-motif peptide (*cyan*) and CSI-motif peptide (*magenta*). CP, capping protein; CPI, CP-interacting; CSI, CARMIL-specific interacting.

The CPI and CSI motifs of the CBR bind to CP at a second site distinct from the site that binds actin and V-1. CARMIL binding to CP induces a conformational change that weakens CP binding to both V-1 and actin. Decreased actin binding results in weaker capping, and this can lead to uncapping of CP-capped barbed ends (15, 16). The effect on actin binding is large, but not complete, and the CARMIL/CP complex retains some level of capping ability (16). The lower level of capping activity is likely to be relevant in cells, considering the concentrations of CP and barbed ends in cytoplasm (9).

CARMIL binding also weakens the interaction of CP with V-1, reflected in an increased rate of dissociation of V-1 from CP/V-1 complex (17, 18). Cytoplasm contains substantial concentrations of V-1 and CP (microMolar range). The two proteins diffuse freely, and their binding affinity is tight (nanoMolar range of *K*_*d*_). Thus, nearly all cytoplasmic CP should be complexed with V-1 and thereby inhibited for binding actin and capping barbed ends. CARMILs and other CPI-motif proteins are present in relatively small amounts, and they are usually found at discrete membrane locations. CARMIL CBR binding to CP affects both the actin-binding site of CP and the V-1–binding site of CP because they overlap with each other (9, 10, 17, 18, 19, 20, 21, 22). Taken together, the net effect of CARMIL on CP in cells is to counteract the inhibitory action of V-1 and thereby activate CP for capping at specific sites on membranes (9, 23). Activated CP is thus available to either promote Arp2/3-based actin assembly or to curtail the growth of free barbed ends, both of which occur near membranes. This model for CP regulation was developed by Fujiwara, Hammer and colleagues (9, 15) based on biochemical observations of stoichiometric inhibition of CP by V-1, activation of CP by CARMIL binding leading to dissociation of the CP/V-1 complex, measurements of CP and V-1 concentrations in cytoplasm, and subcellular localization of the proteins.

The CPI motif binds to the stalk region of the mushroom-shaped CP molecule, as does the C-terminally adjacent CSI motif (10) (see Fig. 1*B*). The linker between the CPI and CSI motifs is not involved in binding to or regulation of CP (24). The third portion of the CBR is the MB domain with its BH motif. The BH motif was discovered and defined using a sequence-search algorithm designed to identify short regions of basic and hydrophobic residues that bind to acidic lipid membranes (25). That study identified BH motifs in mouse CARMIL1, several myosin-I’s, and several PAK kinases (25). The MB domain is present in the conserved sequences of all three vertebrate CARMIL isoforms (26), and each of the three is able to target GFP to the plasma membrane. The MB domain of the human CARMIL2 isoform was found to be necessary for the cellular functions of CARMIL2 in assays related to actin-based motility in cultured cells (26).

A previous study from our lab (27) utilized a fragment of the CBR of human CARMIL1 termed “CBR115,” which contains the CPI and CSI motifs. CARMILs appear to function at membranes, and the MB domain with its BH motif is always in close proximity directly C-terminal to the CPI and CSI motifs. We asked how the MB domain and its binding to lipids affect the regulation of CP, using a single CARMIL1 CBR fragment encompassing all three motifs. We examined how the MB domain affects the binding of CP to actin using functional assays for actin assembly, and conversely, how the interaction with CP affects the ability of a CARMIL fragment to bind to lipids.

We found that the MB domain is sufficient for targeting the CARMIL fragment to an acidic phospholipid membrane and that the targeted CARMIL fragment is able to promote Arp2/3-mediated actin assembly by promoting V-1 dissociation from CP/V-1 complex. Conversely, we found that binding of CP to the CARMIL CBR fragment leads to partial dissociation of the CBR fragment from the lipid. This unexpected observation provides insight into how CARMILs might leave a membrane, and it provides a potential mechanism for the dissociation of CP from actin filament barbed ends, which is strongly suggested by the studies of CP/actin association lifetimes in cells (28, 29).

## Results

The CARMIL family of proteins is distinct among vertebrate CPI-motif proteins because CARMILs contain two conserved regions following the CPI domain—the CSI domain and the MB domain (Fig. 1*A*). The CPI and CSI motifs were defined by structural analysis showing their direct interactions with CP, their sequence conservation, and their biochemical and cellular effects on CP function (9, 10, 22, 24). Previous studies focused on the CPI and CSI domains. Here, we investigated the biochemical functions of the MB domain using a fragment of the CBR of CARMIL1, termed “CBR126,” which includes all three of the CBR domains. First, we examined how the MB domain affects the capping activity of the CPI/CSI domain construct. Second, we examined how the interaction of the MB domain with lipids affects CP activity, in terms of a) capping actin barbed ends and b) promoting Arp2/3-nucleated actin assembly. For (a), we used traditional pyrene-actin capping assays in solution, and for (b), we employed a bead surface-based actin reconstitution system employed in our recent study (27) with the modification of adding anionic phospholipids to the bead surface to mimic the plasma membrane.

**Figure 2:**
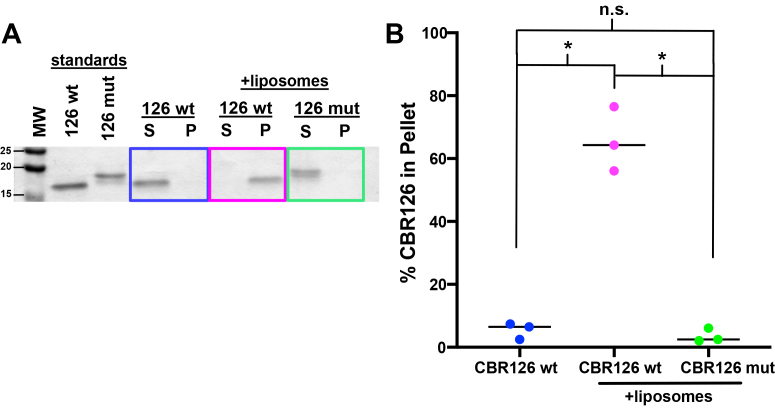
**CBR126 associates with phospholipid membranes *via* the membrane-binding (MB) domain.***A*, SDS-PAGE of protein from the supernatant (S) and pellet (P) following centrifugation in liposome sedimentation assays. CBR126 wt pellets almost completely with liposomes (*magenta*), whereas the membrane-binding mutant CBR126 mut is primarily in the supernatant (*green*). *B*, the percentage of CBR126 in the pellet is plotted as compared to a standard sample loaded on the same gel, in (*A*). Each point is a separate experiment. The *horizontal* line is the median. *Asterisk* denotes *p* < 0.01 in a Welch’s *t* test (41). CBR, CP-binding region.

To begin, we confirmed that the MB domain can target CARMIL1-CBR126 to a lipid membrane, as previously shown in cells (26), using a biochemical assay of liposome sedimentation. CARMIL1-CBR126 sedimented almost completely in the presence of liposomes, and it did not sediment in the absence of liposomes (Fig. 2, *A* and *B*, blue *versus* magenta). This result confirms the prediction, from studies of GFP-MB fusion proteins expressed in cells (26), that the MB domain is sufficient for targeting to a biochemical lipid membrane.

We next asked how the association of CBR126 with a lipid membrane affects its ability to interact with CP and regulate actin assembly. As a negative control for membrane binding of CBR126, we designed and constructed loss-of-function membrane-binding mutants of the MB motif. We changed basic and hydrophobic amino acids known to contribute to the membrane-binding function (changing basic residues to acidic ones and hydrophobic residues to hydrophilic ones) (Fig. 1*A*) (25, 26). First, we mutated the 13-amino-acid core of the BH motif in CARMIL1, previously found to be necessary for CARMIL2 membrane targeting (26). This mutation decreased the association of CBR126 with lipids by approximately half in the liposome sedimentation assay (Fig. S1). We then made a longer mutation of the MB domain, covering a span of 29 amino acid residues (Fig. 1A). This longer mutation greatly decreased, to near zero, the amount of CBR126 pelleting with liposomes in the sedimentation assay (Fig. 2, *A* and *B*, magenta *versus* green). Thus, the residues of the BH motif were confirmed to be responsible for the membrane-binding ability of CBR126. In subsequent experiments testing the functional roles of the MB domain, we compared the activity of CBR126 wt with the longer 29-aa mutant, hereafter termed “CBR126 mut.”

In addition, we deleted the entire MB domain by truncating CBR126 between the CSI and MB domains, creating a fragment termed “CBR81,” to address the question of whether the MB domain has a negative or positive effect on the CPI/CSI interaction with CP. CBR81 did not pellet with liposomes in the sedimentation assay to any appreciable degree (Fig. S1), again showing the necessity of the MB region for interaction of the CBR region of CARMIL1 with lipids.

### Effect of MB domain on actin capping activity in solution

**Figure 3:**
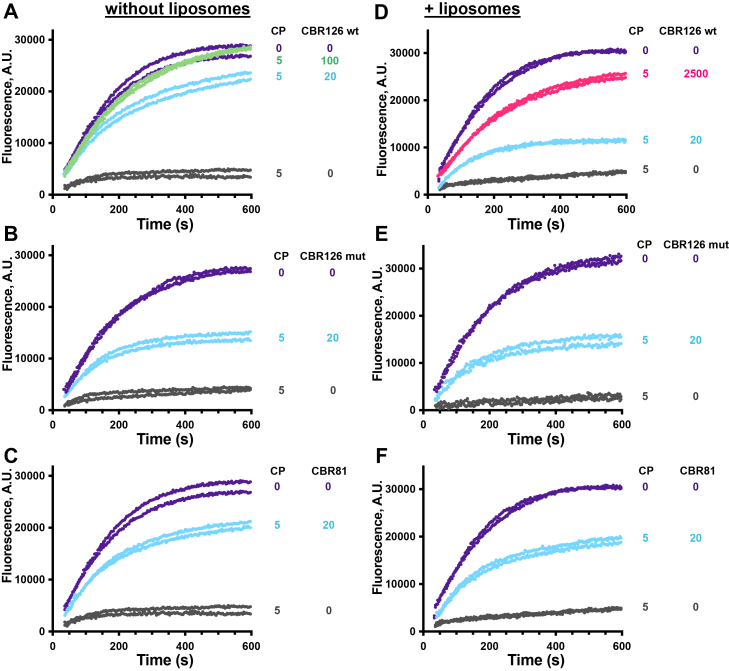
**Effects of CBR126 MB domain and lipids on actin capping activity of CP.** Pyrene actin polymerization assays seeded with barbed ends performed in the absence (*A* to *C*) and presence (*D* to *F*) of liposomes. Each curve is one independent experiment. Concentrations of CP and CBR fragments (nM) as indicated. *A*, CBR126 wt inhibits CP (*blue* and *green* curves). *B*, similar to (*A*), with CBR126 MB mut. *C*, similar to (*A*), with CBR81. *D* to *F*, analogous to (*A* to *C*), with the addition of liposomes. Far more CBR126 wt (125×) is needed to inhibit CP (compare *pink* curves in (*D*) with *blue* curves in (*A*). *E* and *F*, liposomes have no apparent effect on the activity of CBR126 mut or CBR81. NB: These curves are selected from a complete titration set of curves shown in Fig. S2. CBR, CP-binding region; CP, capping protein; MB, membrane binding.

Next, we asked how the MB domain affects the ability of the CBR to regulate actin capping activity of CP in solution, using pyrene-actin polymerization assays. Increasing concentrations of CBR126 wt inhibited the actin capping activity of CP (Figs. 3*A*, and S2 for full titration curves). The CBR126 mutant also inhibited CP well, but not as effectively as the CBR126 wt (Fig. 3, *A* *versus* *B*, blue curves). The binding affinities of CBR126 wt and CBR126 mut for CP, measured by isothermal calorimetry (ITC), were similar (Fig. S3). We also tested the truncated CARMIL1 CBR81 fragment, lacking the MB domain. CBR81 was about as effective as CBR126 wt at inhibiting CP in the actin capping assay (Fig. 3, blue curves of *A* and *C*).

Since the MB domain is always located just C-terminal to the CPI and CSI motifs in CARMILs, we hypothesized that membrane interaction might affect the ability of CPI/CSI to interact with CP. To investigate whether and how lipid binding affects the interaction activity of CBR126 with CP, we added lipid membranes in the form of liposomes to the pyrene-actin–based capping assays. When liposomes were present, 125× more CBR126 wt was required to obtain the same level of CP inhibition as when liposomes were absent from the assay mixture (Fig. 3, *D* pink curve compared to *A* blue curve). As a negative control, we tested the CBR126 mut with a dose-response assay (Figs. 3 and S2); the results confirm that liposomes do not significantly alter the actin capping activity of the MB mutant (Fig. 3, *E* compared to *B*). As an additional negative control, we tested the activity of CBR81, truncated to lack the MB domain entirely. We found that CBR81 was also not significantly affected by the presence of liposomes (Fig. 3, compare *F* with *C*). Overall, the important conclusion is that the interaction of CBR126 with the liposome membrane decreases the biochemical effect of CBR126 on the capping activity of CP.

To further investigate the biochemical interactions of CBR126 with membranes and CP, we added CP to liposome sedimentation assays. As shown above (Fig. 2), CBR126 sedimented with liposomes. When an equimolar amount of CP was added to the reaction mixture, the amount of CBR126 sedimenting with the liposomes was less, indicating that the interaction of CBR126 with CP decreases the MB of CBR126 (Fig. S4*A*). In a titration experiment, increasing amounts of CP in the mixture led to corresponding decreases in the amount of CBR126 sedimenting with liposomes (Fig. S4*C*) along with decreases in the fraction of CP sedimenting with liposomes (Fig. S4*D*). CP was present in the pellet fraction along with CBR126 (Fig. S4*B*), showing that CP is able to bind to CBR126 that is bound to membrane. Note that CP alone, in the absence of CBR126, did not pellet with liposomes. We conclude that a) CP can bind to membranes *via* its interaction with CBR126, b) the CBR126/CP complex more readily leaves the membrane, because c) CP weakens the interaction of CBR126 with the membrane. In other words, CBR126 can bind to the lipid membrane and to CP. The binding states of CBR126 are dynamic and interconvert; they are not exclusive of one another. The interactions provide a potential mechanism by which CP could be activated at the membrane and then rapidly released from the membrane once attached to and incorporated into the actin filament network.

### Arp2/3-mediated actin assembly at lipid-coated surface

Previously, we demonstrated that CP can be activated by CARMIL1 at a surface by releasing CP from V-1, using a biomimetic bead assay for Arp2/3-mediated actin assembly (27). In that study, an active fragment of CARMIL1 fused to glutathione-S-transferase (GST) was coupled to glutathione-coupled beads. Here, to create a surface that mimics the membrane bilayer, we deposited anionic phospholipid onto a bead. We documented lipid deposition by fluorescence imaging of beads with a fluorescent lipid tracer and by scanning electron microscopy (Fig. S5).

**Figure 4:**
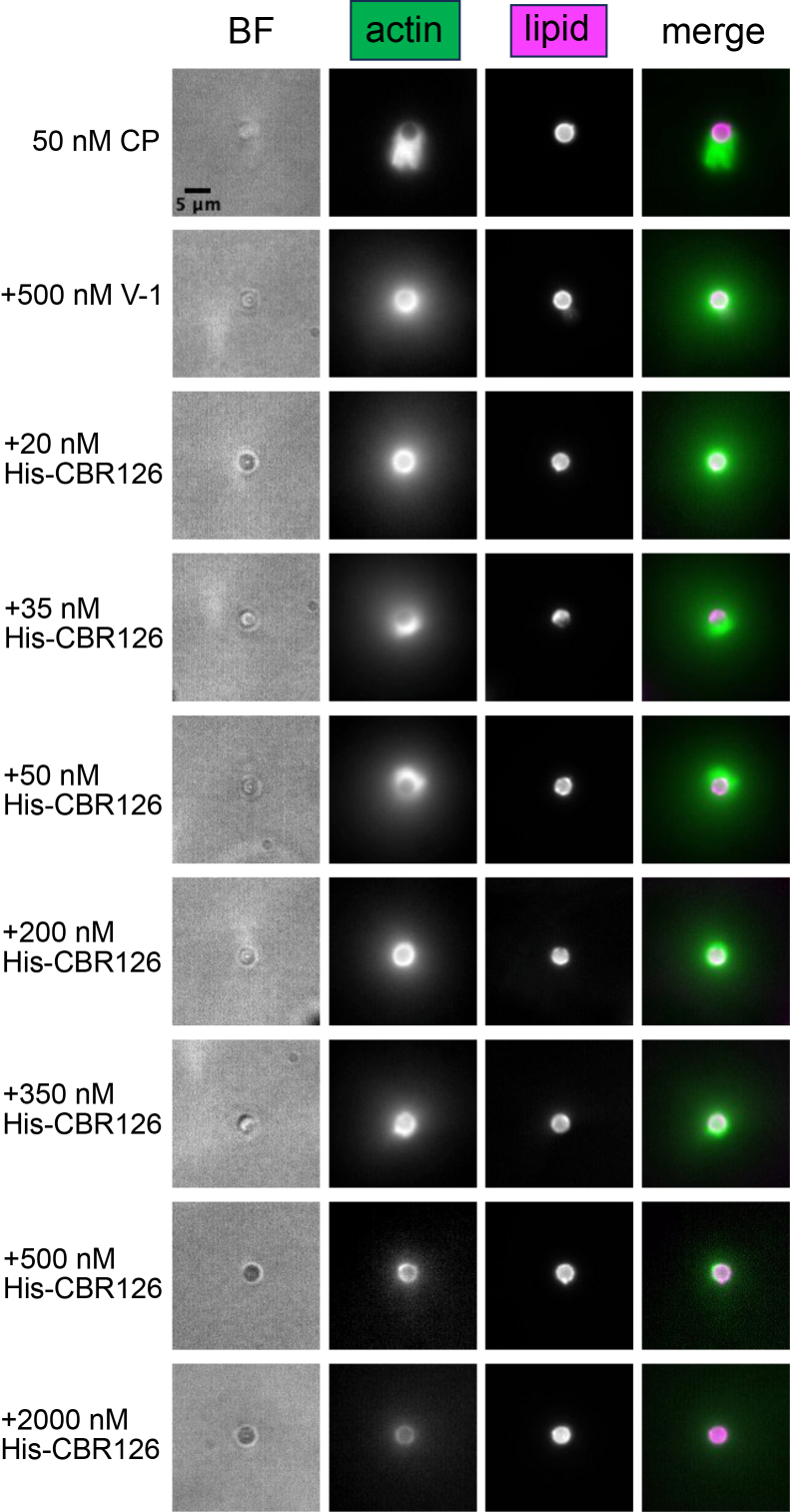
**Actin filament network assembly on lipid-coated beads with CP, V-1, and CBR126.** Asymmetric tails of actin filament networks generated by incubating Ni-functionalized and fluorescent lipid-coated beads with His-VVCA (N-WASP), followed by the addition of 100 nM Arp2/3 complex, 5 μM profilin-actin, and 50 nM CP for 30 min (*top row*). Addition of 500 nM V-1 to the reaction mixture resulted in F-actin growing from the bead surface as a symmetric ring and a diffuse cloud around the bead (*second row*). Addition of low concentrations of His-CBR126 resulted in asymmetric F-actin tail growth from the bead (rows labeled 35 nM and 50 nM), and higher concentrations of CBR126 inhibited actin growth (row labeled 2000 nM). CP, capping protein; CBR, CP-binding region.

First, we asked whether CBR associated with the lipid-coated bead surface is able to activate CP and promote Arp2/3-nucleated actin assembly (27). In our recombinant system, Arp2/3 complex is activated by a VVCA fragment of N-WASp. To couple the VVCA to the bead, we used His-tagged VVCA and Ni-functionalized lipids. In the initial set of experiments, CBR was not included. On addition of Arp2/3 complex and actin, actin polymerization was observed around the bead, in the form of asymmetric tails. The actin assembly required CP (Fig. 4, top row), and addition of V-1 suppressed actin tail growth by inactivating CP (Fig. 4, second row). The results document that Arp2/3-mediated actin polymerization can occur from the lipid-coated beads, that active CP is necessary, and that V-1 inhibits CP and prevents actin growth.

### Activity of CBR126 at lipid-coated bead surface: targeting *via* His tag

We asked whether CBR126 was able to activate CP (by overcoming the inhibition of V-1) and induce Arp2/3-mediated actin assembly at the surface of a lipid-coated bead, as a mimic of a cellular membrane. As a first step, we compared new results here with CBR126 and lipid-coated beads with those from our previous study (27), which used a shorter fragment of the CBR (CBR115), lacking part of the MB domain (27) and which coupled GST-CBR115 to glutathione beads. For this comparison, we targeted CBR126 to the lipid-coated beads using a His-tagged version of CBR126; the His tag was designed to couple CBR126 directly to the Ni-functionalized lipid-coated beads, independently of the MB domain, in order to be analogous to our previous results with GST-CBR115 and glutathione beads. As predicted, the actin assembly results were similar to those of our previous study, specifically that low amounts of His-tagged CBR126 caused asymmetric actin tail growth close to the bead surface, presumably by activating CP from CP/V-1 complex (Fig. 4, 35 nM and 50 nM, rows 4 and 5). With higher amounts of His-CBR126, actin tail growth was inhibited, presumably by the direct effect of CBR126 on the actin capping activity of CP (Fig. 4, 500 nM–2000 nM, rows 8 and 9). The inhibition of CP activity at high concentrations of CBR126 in this assay is consistent with previous studies showing that CARMIL CBR at high levels decreases the actin capping activity of CP in the absence of V-1 (16, 22).

To assess the contribution of the MB domain to the activity of CBR126 on CP and actin network growth in the bead assay without consideration of the MB domain directly targeting CBR126 to the beads, we compared His-tagged CBR126 wt with the MB mutant CBR126 (His-CBR126 mut). As described above, we identified a concentration of His-CBR126 wt that produced a high level of asymmetric actin network growth near the bead surface. Next, we compared His-CBR126 wt with His-CBR126 mut and counted the number of beads with asymmetric actin tail growth. We observed slightly less asymmetric actin growth from His-CBR126 mut beads compared to His-CBR126 wt beads with the same concentrations for His-CBR126 (75% of beads for His-CBR126wt versus 61% of His-CBR126 mut, see Table S1).

**Figure 5:**
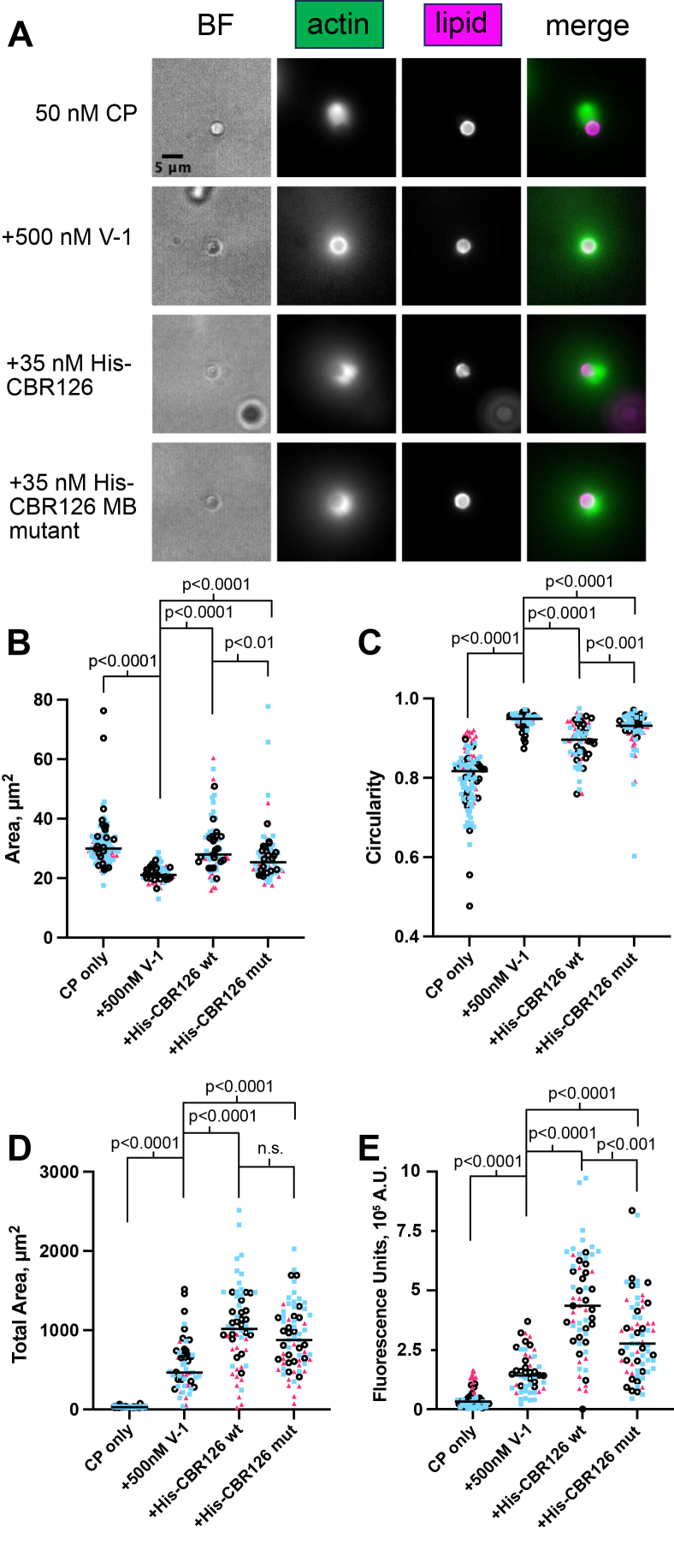
**Membrane-binding domain (MB) effects on CP activity.***A*, His-tagged MB mutants cause actin network to grow asymmetrically from the bead surface in a mixture of 100 nM Arp2/3 complex, 5 μM profilin-actin, 50 nM CP, and 500 nM V-1 (30-min time points) but to a lesser extent than His-CBR126 wt. *B*-*E*, three data sets are plotted as different *colors* and *shapes*. The data analyzed are from experiments with an optimal concentration of His-CBR, one that produced the highest numbers of beads with asymmetric actin growth for each data set. The *horizontal black* bar is the median, and *p* values are calculated from a Mann-Whitney nonparametric analysis. The following parameters were measured and plotted: *B*, the area of brightest fluorescence near the bead surface; *C*, circularity of the region of brightest fluorescence near the bead surface; *D*, the total area of fluorescence, including the diffuse cloud surrounding the beads, and *E*, the total fluorescence of the actin network. CP, capping protein; CBR, CP-binding region.

In addition to counting beads with actin tails, we measured the area and circularity of the brightest region of actin fluorescence signal immediately surrounding the bead. Compared to actin growth on beads in the presence of V-1, beads with His-CBR126 wt had a larger area of brightest fluorescence near the bead surface and a lower circularity, indicating more asymmetric actin growth (Fig. 5, *A*, compare rows 2 and 3, and *B* and *C*). Compared to the wt beads, the mutant CBR126 (His-CBR126 mut) produced less area and greater circularity (Fig. 5*A*, compare rows 3 and 4, and *B* and *C*). Also, we measured the overall fluorescence of the actin network. Both wt and mutant His-CBR126 increased the overall fluorescence of the actin network (relative to V-1 alone), but the increase observed for the mutant CBR126 (His-CBR126 mut) was less than that for the WT CBR126 (His-CBR126 wt) (Fig. 5*E*). This result for mutant versus WT is consistent with the results of the pyrene actin polymerization assays, in which the MB mutant form of His-CBR126 exhibited biochemical activity towards CP, but at a level less than that of the WT form of His-CBR126 (compare Figs. 3, *A* and *B* and S2, *A* and *B*). We also measured the total area of the diffuse actin cloud surrounding the bead. For this parameter, there was no significant difference between WT and mutant forms of His-CBR126; the values for both WT and mutant forms were increased by similar extents, relative to V-1 alone (Fig. 5*D*). This significance of this point will be discussed in the Discussion section, drawing a comparison to the results from the set of experiments presented next, in which CBR126 is targeted by its MB domain, not a His tag.

Taken together, we conclude that, when His-tagged CARMIL1 CBR126 is targeted to the lipid-coated bead surface *via* its tag, the MB domain does contribute to the biochemical activity of the CBR toward CP, complementing the effects of the CPI and CSI motifs. The MB domain does influence CP actin capping activity and thus actin network assembly.

### Activity of CBR126 at lipid-coated bead surface: Targeting *via* MB domain

Having demonstrated that CBR126 targeted to the lipid-coated beads surface *via* a His tag supports the activation of CP and Arp2/3-mediated actin assembly, confirming our previous results with CBR115 targeted *via* GST and glutathione, we next asked whether the MB domain of CBR126 was sufficient to target CBR126 to the beads on its own and whether CBR126 bound *via* its MB domain was able to activate CP and promote actin assembly. First, we prepared CBR126 lacking the His-tag in order to ask whether the MB domain of CBR126 was sufficient to target CBR126 to the lipid-coated beads. To assess recruitment of CBR126 to the beads, we fluorescently labeled CBR126 lacking a His tag and followed its localization with fluorescence imaging. Fluorescent CBR126 appeared on the surface of the lipid-coated bead, prior to adding the actin assembly mixture (Fig. S6, *A*, top row, and *B*), indicating that the MB domain of CARMIL1 CBR126 is sufficient for localizing CBR126 to the lipid-coated bead surface.

**Figure 6:**
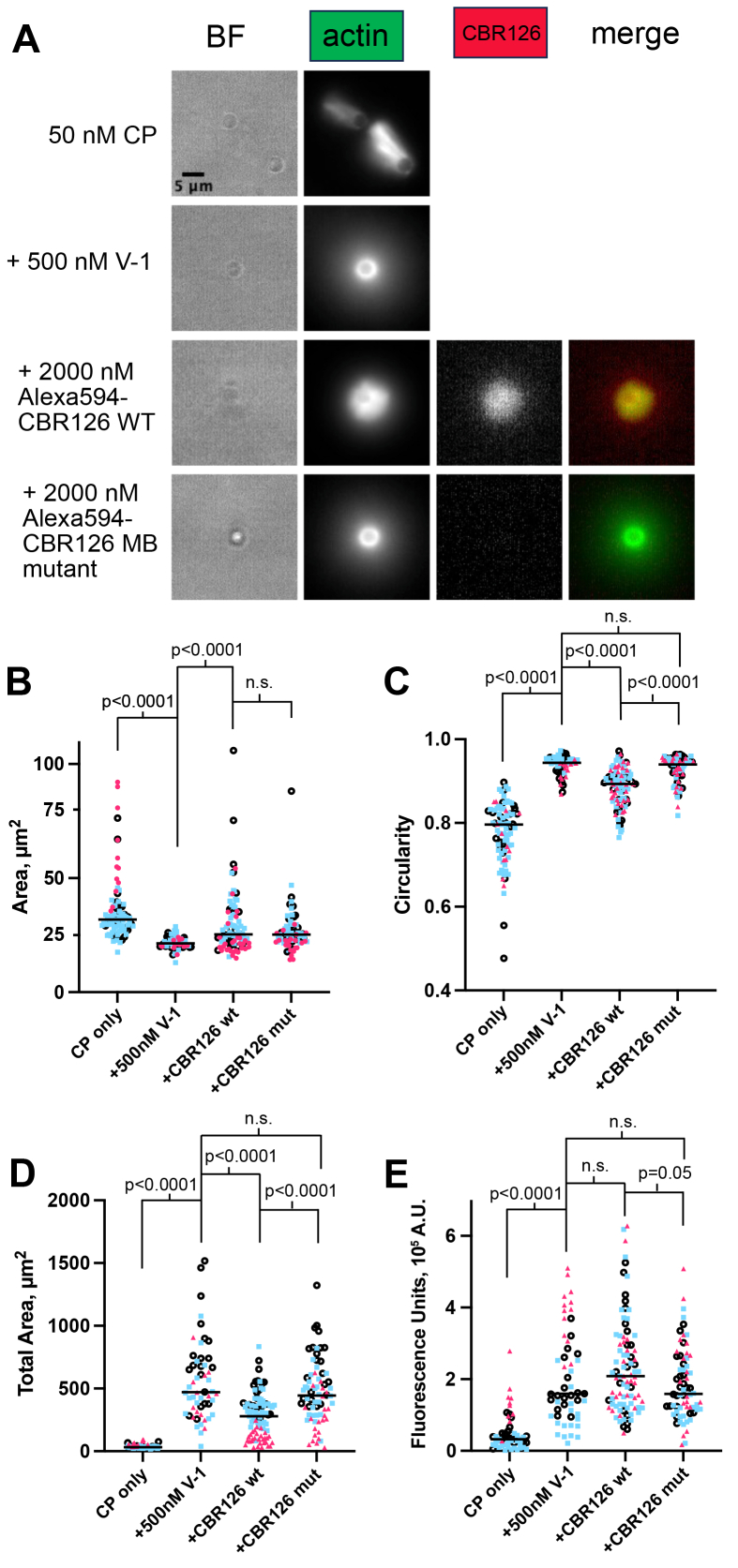
**Membrane-binding domain (MB) important for function because of CARMIL localization.***A*, untethered CBR126 associates with the lipid beads *via* the MB domain and promotes asymmetric actin growth. MB mutants do not associate with the lipid beads and do not show the same effects on the actin network. *B–E*, three data sets are plotted as different *colors* and *shapes*. The data analyzed is the concentration of untethered CBR which produced the highest numbers of beads with asymmetric actin growth for each data set. The *horizontal black* bar is the median, and *p* values are calculated from a Mann-Whitney nonparametric analysis. The following parameters were measured and plotted: *B*, the area of brightest fluorescence near the bead surface; *C*, circularity of the region of brightest fluorescence near the bead surface; *D*, the total area, including the diffuse actin cloud surrounding the beads; and *E*, the total fluorescence of the actin network. CBR, CP-binding region.

Next, we asked whether CBR126 bound by its MB domain to the surface of the lipid-coated bead was sufficient to activate CP and promote Arp2/3-induced actin assembly. After adding all the components of the actin assembly reaction mixture, we observed robust and asymmetric actin growth on beads (Fig. 6, panel *A*, row 3).

In the pyrene actin polymerization assays, discussed above, the effect of CBR126 on the activity of CP was decreased when liposomes were present. We observed that lipid binding to CBR126 had a similar effect here, in the bead surface assay. In pilot experiments to define optimal conditions for actin assembly, the concentrations of CBR126 without His tag and targeted *via* the MB domain required to produce Arp2/3-mediated actin assembly were larger than the amounts of CBR126 required when targeted *via* the His tag. As evidence, the optimal concentrations of His-tagged CBR126 utilized in Figure 4 were 35 nM and 50 nM, while the optimal concentrations of nontagged CBR126 utilized in Figure 6 were 500 nM and 2000 nM.

### Localization of nontagged CBR126 during the actin assembly reaction

The binding of the CBR126 MB domain to the membrane is based on nonspecific ionic and hydrophobic interactions with the acidic phospholipids of the membrane, suggesting that the dissociation rate might be appreciable. To investigate that possibility, we asked how the location of CBR126 changed as the actin polymerization reaction progressed. Over a period of time (30 min), the intensity of CBR126 fluorescence on the bead surface decreased, indicating that CBR126 left the bead surface (Fig. S6, *C* compared to *A*). For some of the beads, the remaining CBR126 fluorescence overlapped with the fluorescence of the diffuse filamentous actin (F-actin) cloud network, based on two-color imaging (Fig. 6*A*, row 3). We interpret this result to mean that CBR126 moved away from the surface with the growing actin network, presumably binding to CP attached to barbed ends of actin filaments. For other beads, no detectable CBR126 fluorescence was observed, either with the actin network or at the bead surface. We complemented this approach by localizing CBR126 using immunofluorescence with anti-CBR126 antibodies. The results were similar, confirming that CBR126 left the bead surface and was present, in some cases, distributed throughout the actin network (Fig. S6, panel *D*).

For the beads that did not have any associated fluorescence, one can speculate that the fluorescence intensity was below the sensitivity of the microscope system. Alternatively, the CBR126 may have detached from the actin filament network and diffused away; this would be expected if CBR126 attached to CP induced uncapping of the barbed end, followed by diffusion of CBR126 and its associated CP away from the actin filament network.

### Functional effects of the MB domain on actin assembly: MB wt versus mutant

To confirm that the MB domain is responsible for the lipid membrane localization and for the functional effects on actin assembly, we compared nontagged WT CBR126 with the MB mutant form of CBR126. First, we observed the localization of CBR126 directly using a fluorescent derivative, as above. The WT form of CBR126 was observed on the lipid-coated bead surface prior to adding the actin assembly reaction mixture, as noted above; however, only a small amount of the MB mutant form of CBR126 was observed at the bead surface (Fig. S6, *A* and *B*). The residual localization might result from the fact that the mutation changed part but not all of the BH motif residues (Fig. 1). This result is consistent with the liposome sedimentation assays, where pelleting of the CBR126 mutant with liposomes was greatly decreased but not completely abolished.

We predicted that the nearly complete lack of targeting of the MB mutant would lead to a loss of functional effects on CP activity and actin assembly. Actin assembly was induced with beads to which mutant CBR126 was added. As expected, the percentage of beads with asymmetric actin assembly was substantially less for mutant CBR126 compared to WT (78% of the CBR126 wt beads versus 25% of the CBR126 mut beads, Table S2; representative images shown in Fig. 6*A*, bottom two rows). In addition, the circularity parameter was increased (*i.e.* the actin assembly was less asymmetric) for CBR126 mutant versus WT (Fig. 6*C*).

The difference between WT and mutant was also apparent in the total area of the diffuse actin clouds surrounding the beads. Compared to the control with V-1 alone, the area was decreased with the addition of WT CBR126, and this change in the organization of the actin clouds did not occur with CBR126 mut (Fig. 6*D*). Interestingly, after actin growth for 30 min, we did observe some recruitment of CBR126 mut to the diffuse actin network, presumably *via* its CP-binding domains (CPI and CSI), but not to the extent seen with CBR126 wt (Fig. 6*B*).

In sum, the results show the following: a) the MB domain of CARMIL1 CBR126 is necessary and sufficient for localizing CBR126 to a lipid membrane; b) CBR126 targeted to a lipid membrane by its MB domain is able to bind and activate CP and thus promote Arp2/3-mediated actin assembly; and c) CBR126 can transition off the lipid-coated bead surface when CBR126 binds to CP and the dendritic actin network assembles, due to the dynamic nature of the interaction of the BH motif with lipid.

## Discussion

CARMILs bind directly to CP and allosterically decrease its ability to bind V-1 and cap actin filaments (9). The CBR of CARMILs is an ∼130-aa segment of the intrinsically disordered C-terminal half of the ∼1400-aa polypeptide. From N- to C-term, the CBR consists of three domains in tandem: the CPI domain, the CSI domain, and the MB domain. The CPI and CSI domains bind directly to the surface of CP, and they induce a conformational change in CP that affects its distant binding sites for F-actin and V-1.

The conserved position of the MB domain within the three-part tandem array of the CBR raises questions of how the MB domain itself or the binding of the MB domain to a lipid membrane might affect the interaction of the CBR with CP. The MB domain is necessary for the function of CARMIL2 in human cultured cells (26), and the MB domain of any one of the three vertebrate CARMIL isoforms is sufficient to target intracellular GFP to cell membranes (26).

We asked how the MB domain and lipids affect the ability of the CPI and CSI domains to regulate the biochemical activity of CP in the assays of actin capping and Arp2/3-mediated actin nucleation. First, we found that the MB domain is sufficient to target the CBR to a lipid membrane, in binding assays with liposomes or lipid-coated beads. Second, we found that the CBR, when targeted to a lipid-coated bead, is able to activate CP (by inhibition of V-1 binding) and thus promote actin assembly nucleated by Arp2/3 complex. Third, we found that lipid binding decreases the ability of CBR to inhibit the actin capping activity of CP in solution, based on pyrene-actin polymerization assays with liposomes and in Arp2/3-induced actin assembly at a bead surface.

The interaction of the MB domain with a lipid membrane is a dynamic one based on a set of relatively weak nonspecific interactions of basic and hydrophobic residues with the acidic phospholipids of the membrane. We found that CBR126 partitions between the membrane and the soluble phases and that the binding of CP increases the fraction of CBR126 that leaves the membrane for the soluble phase.

Together, these observations indicate that CBR that dissociates from the lipid membrane is relatively more effective in binding CP than is membrane-bound CP. Greater binding of CBR to CP implies greater activation of CP, based on the dissociation of CP/V-1 complex, and it implies greater inhibition of CP’s capping of the barbed ends of actin filaments. Both of these effects, dissociation of V-1 and inhibition of capping, are expected to promote actin assembly near the membrane, but for different reasons. Dissociation of V-1 increases free CP and thereby Arp2/3-mediated actin nucleation. Inhibition of capping by CP should increase the growth of barbed ends created by any mechanism, not just Arp2/3-based nucleation.

The dynamic nature of the MB domain/membrane interaction was observed in both the liposome sedimentation experiments and the lipid-coated bead-based actin assembly reconstitution system in this study. First, in liposome sedimentation experiments, a fraction of CP sedimented with liposomes but only when CBR was present, as predicted. CBR with a mutated form of the MB, lacking basic and hydrophobic residues, was not able to bind liposomes and thus did not suffice to recruit CP to the liposomes. More important, when CP was present and bound to CBR, then the fraction of CBR associated with the liposomes was decreased.

Second, in the lipid-coated bead-based actin assembly reconstitution experiments, nontagged CBR was initially associated completely with the bead surface. However, as the actin filaments assembled and grew away from the bead surface, the CBR was observed to leave the bead surface. A fraction of CBR was found associated with the actin filament network that grew from the bead surface. One interesting functional consequence of CBR leaving the membrane was that the total area of the assembled actin network including the diffuse actin cloud was smaller for the nontagged CBR (bound *via* its MB domain) than for His-tagged CBR, which did not leave the bead surface.

The resulting morphology of the actin assembled around the bead included several striking observations with potentially important conclusions. First, in control experiments with CP alone (without V-1 or CBR), the assembled actin consisted only of asymmetric tails of F-actin, not diffuse clouds. Addition of V-1, to inhibit CP, produced large diffuse clouds of F-actin with few or no asymmetric tails. Adding His-tagged CBR126, which remained at the bead surface, led to asymmetric tail formation with large diffuse clouds. With untagged CBR126, targeted *via* its MB domain, the actin produced asymmetric tails with much smaller diffuse clouds. These effects are consistent with CBR continuing to activate CP (by inducing dissociation of CP/V-1 complex) after CBR leaves the membrane. This conclusion is also suggested by the observations that MB-containing CBR interacts more strongly with CP than does either the MB-less (CBR81) or the MB mutant forms of CBR. Therefore, we speculate that CBR that leaves the membrane continues to affect actin assembly, by activating CP or by uncapping barbed ends.

### Model for CARMIL/CP function at membranes

**Figure 7:**
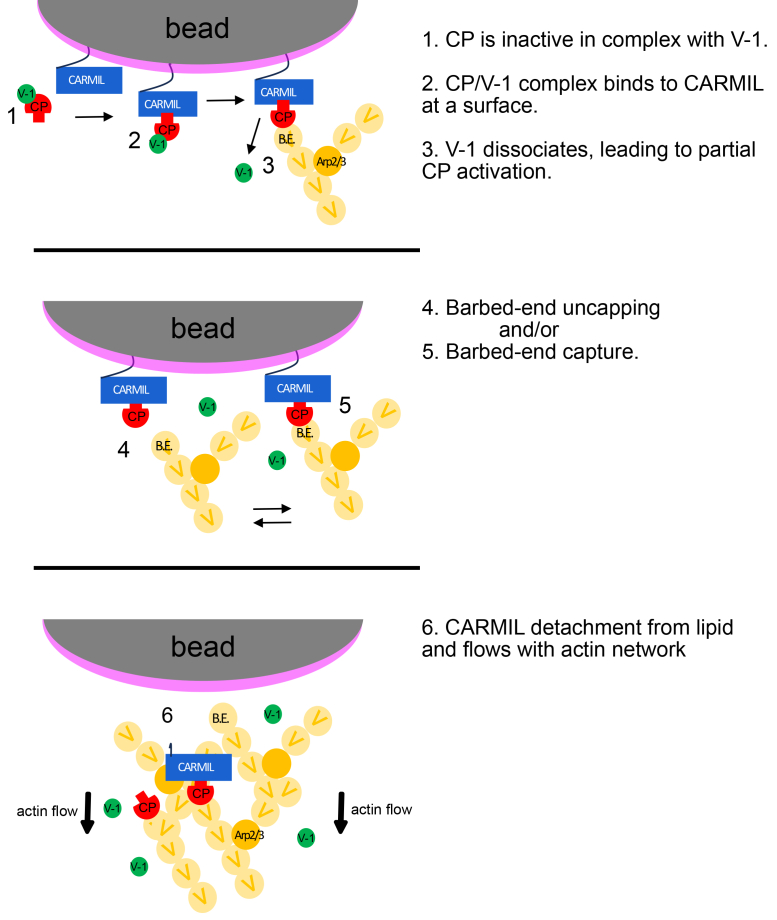
**Model of regulatory cycles for CP actin capping.***1*, CP bound to V-1 in the cytoplasm is inactive. *2*, CP/V-1 binding to CARMIL promotes V-1 dissociation. *3*, Free CP binds barbed ends and promotes Arp2/3-nucleated polarized actin growth at the bead surface. *4 & 5*, Near the bead surface, CARMIL can a) promote uncapping of a capped barbed end to allow filament growth or b) capture a capped actin filament. Dynamic association of CP with barbed end - “loose/leaky” capper. *6*, Dynamic association of CARMIL with lipid: CARMIL can leave the bead surface and stay bound to CP as the actin filament network grows and flows away from the bead surface. CP, capping protein.

Together, these observations substantiate the existence of multiple states for CARMIL and CP. These states and their functional properties have been proposed to account for features of actin assembly observed in the studies of actin-based cell motility. One can envision multiple paths between the states based on the biochemical results in this study. These biochemical states may correspond with the states of actin filament membrane attachment and actin-based assembly in cells. The biochemical states and the paths between are illustrated, at least in part, in the cartoon model of Figure 7.

In the cartoon model, CARMIL is sufficient to bind CP to a membrane and to activate CP for barbed-end capping (Fig. 7). Membrane-bound CARMILs may thereby contribute to the morphological appearance of actin filament barbed ends described as attached to or embedded in electron-dense material adjacent to the plasma membrane (30, 31, 32). Second, CARMILs can convert capped barbed ends to uncapped ones, as documented previously with single-molecule biochemical experiments (15). Also known from previous studies, active CP near the membrane suffices to promote Arp2/3-based actin assembly from new free barbed ends (33, 34). The dynamic nature of the CBR MB/membrane interaction observed here implies that CARMIL is able to leave the membrane in cells (Fig. 7). Once off the membrane, the ability of the CBR to promote uncapping (dissociation of CP from the barbed end) is expected to increase, based on these and previous biochemical results. Finally, uncapping is more likely to occur during retraction of a cell process, when CARMIL is no longer bound to the membrane (23, 35).

### Open questions and future directions

First, this study utilized only the CBR fragment of CARMIL, not full-length protein. The field recognizes that other regions of CARMILs, outside the CBR, have other biochemical functions related to interactions with other biomolecules and to the regulation of CARMIL activity. For CARMIL1, studies of membrane localization have implicated the noncanonical PH domain near the N terminus and the homodimerization domain as having potential roles (13). In addition, evidence exists that CARMILs can be autoinhibited (14, 36), which may be regulated by signaling molecules (9).

Important future directions for the field will include defining the relative abundance of the states considered and detected here, along with measuring the relative timing and rate of transitions of CARMILs and CP among those states.

## Conclusions

The MB domain of CARMIL is sufficient to attach the CBR to lipids. On the surface of a lipid-coated bead, CBR attached *via* its MB domain is able to activate CP and promote Arp2/3-mediated actin assembly.

CBR on the membrane, bound *via* its MB domain, is less active for CP interaction compared to CBR in solution.

CBR interaction with lipids is dynamic, converting between lipid-bound and free in solution. The transition of CARMILs between states on and off the membrane may help account for the attachment of actin filament barbed ends to the membrane, as well as for detachment and uncapping. These transitions and states may correspond to ones that occur at the leading edge of motile cells.

## Experimental procedures

Many of the Methods are similar to ones in a previous publication (27). Modifications to procedures are stated explicitly.

### Buffers

Assay buffer: 20 mM Hepes, 100 mM KCl, 1 mM MgCl_2_, 1 mM EGTA, 1 mM Tris(2-carboxyethyl)phosphine (TCEP), 1 mM ATP (pH 7.0). For bead actin polymerization fluorescence microscopy assays, the buffer included 0.2% methylcellulose (cP 400) to reduce Brownian motion, 2.5 mg/ml bovine serum albumin to decrease nonspecific sticking to the coverslip, and 20 mM beta-mercaptoethanol to lessen photobleaching.

### Proteins

#### Actin, Arp2/3 Complex, Profilin, CP, V-1, and CARMIL1 CBR

Unlabeled, pyrene-labeled, and Alexafluor 488–labeled gel-filtered G-actin from skeletal muscle α-actin (UniProt P68135, *Oryctolagus cuniculus*) were prepared as described (27). Porcine brain Arp2/3 complex from cytoskeleton (Cat. No. RP01P) was reconstituted per manufacturer instructions and used within 1 month. Human profilin-1 (UniProt P07737) was a gift from Dr Silvia Jansen (Department of Cell Biology & Physiology, Washington University, St Louis). Mouse CPα1β2 heterodimer (UniProt Q5RKN9 and Q923G3, pBJ 2041) and human V-1 (UniProt P58546, pBJ 2438) were purified as described (37).

CARMIL1 CBR126 WT (pBJ 2491) was composed of MVKIH, a translational enhancement sequence (38), followed by HHHHH (His tag), LEVLFQGP (PreScission protease cleavage site), and human CARMIL1 (UniProt Q5VZK9) E964 to T1089. Codon-optimized DNA was synthesized and inserted into a pRSF-1b vector (Novagen) by Azenta Life Sciences. Recombinant protein was expressed in *Escherichia coli* NiCo21 (DE3) (New England Biolabs). Cells were lysed, and the insoluble fraction of the lysate was dissolved in 20 mM sodium phosphate, 2 mM TCEP, 1 mM NaN3, containing 6 M guanidine–HCl (pH 7.8). The protein was bound to Ni-NTA 6 Fast Flow (Cytiva). Guanidine–HCl was washed from the Ni-NTA column with 20 mM Tris–HCl, 0.5 mM TCEP, 0.3 M NaCl, 1 mM NaN3 (pH 7.8), and the CARMIL peptide was eluted with 20 mM Tris–HCl, 0.5 mM TCEP, 0.3 M NaCl, 1 mM NaN3, 100 mM imidazole (pH 7.8). To prepare the tag-less CARMIL1 peptide, the 6x-His tagged peptide was cleaved with GST-tagged PreScission protease (Cytiva). The 6x-His tagged and tag-less peptides were each then purified by cation exchange chromatography using a POROS GoPure XS column (Applied Biosystems) in 20 mM sodium phosphate, 0.1 mM EDTA, 1 mM NaN3, 10 mM DTT (pH 7.5), eluted with a linear gradient to 1 M NaCl. Fractions containing purified peptide were pooled, dialyzed against 20 mM 3-(N-morpholino)propane-sulfonic acid (Mops), 100 mM KCl, 1 mM TCEP, 1 mM NaN3 (pH 7.2), and stored at −70 °C. Molar mass of the purified peptides was confirmed by MALDI mass spectrometry.

CARMIL1 CBR126 mut (pBJ 2549) preparation was similar to the CBR126 wt above (pBJ 2491) with several differences. The expressed construct was composed of MVKIH, a translational enhancement sequence (38), followed by HHHHH (His tag), LEVLFQGP (PreScission protease cleavage site), and a mutated version of human CARMIL1 (UniProt Q5VZK9) E964 to T1089 with replacement of the MB domain with the mutated sequence EEEEDSEESSGSSNSSESESESEEPPTISMT. Codon-optimized DNA was synthesized and inserted into a pRSF-1b vector (Novagen) by Azenta Life Sciences. Recombinant protein was expressed in *E. coli* NiCo21 (DE3) (New England Biolabs). Cells were lysed, and the insoluble fraction of the lysate was dissolved in 20 mM sodium phosphate, 2 mM TCEP, 1 mM NaN3, containing 6 M guanidine–HCl (pH 7.8). The protein was bound to Ni-NTA 6 Fast Flow (Cytiva). Guanidine–HCl was washed from the Ni-NTA column with 20 mM Tris–HCl, 0.5 mM TCEP, 0.3 M NaCl, 1 mM NaN3 (pH 7.8), and the CARMIL peptide was eluted with 20 mM Tris–HCl, 0.5 mM TCEP, 0.3 M NaCl, 1 mM NaN3, 100 mM imidazole, containing 6 M urea (pH 7.8). The CARMIL peptide was then purified by anion exchange chromatography using a POROS GoPure XQ column (Applied Biosystems) in 20 mM Tris–HCl, 1 mM NaN3, 10 mM DTT, containing 6 M urea (pH 7.5), eluted with a linear gradient to 1 M NaCl. Fractions containing purified peptide were pooled and dialyzed against 20 mM Mops, 100 mM KCl, 1 mM TCEP, 1 mM NaN3 (pH 7.2). To prepare the tag-less CARMIL1 peptide, the 6x-His–tagged peptide was cleaved with GST-tagged PreScission protease (Cytiva) The GST-tagged PreScission protease was removed using Glutathione Superflow Agarose (Pierce Chemical Company). The peptides were stored at −70 °C. Molar mass of the purified peptides was confirmed by MALDI mass spectrometry.

CARMIL1 CBR81 WT (pBJ 2531) was composed of MVKIH, a translational enhancement sequence (38), followed by HHHHH (His tag), LEVLFQGP (PreScission protease cleavage site), and human CARMIL1 (UniProt Q5VZK9) E964 to T1044. Codon-optimized DNA was synthesized and inserted into a pRSF-1b vector (Novagen) by Azenta Life Sciences. Recombinant protein was expressed in *E. coli* NiCo21 (DE3) (New England Biolabs). Cells were lysed, and the insoluble fraction of the lysate was dissolved in 20 mM sodium phosphate, 2 mM TCEP, 1 mM NaN3, containing 6 M guanidine–HCl (pH 7.8). The peptide was bound to Ni-NTA 6 Fast Flow (Cytiva). Guanidine–HCl was washed from the Ni-NTA with 20 mM Tris–HCl, 0.5 mM TCEP, 0.3 M NaCl, 1 mM NaN3 (pH 7.8) and the CARMIL peptide was eluted with 20 mM Tris–HCl, 0.5 mM TCEP, 0.3 M NaCl, 1 mM NaN3, 100 mM imidazole (pH 7.8). The protein was then purified by cation exchange chromatography using a POROS GoPure XS column (Applied Biosystems) in 20 mM sodium phosphate, 0.1 mM EDTA, 1 mM NaN3, 10 mM DTT (pH 7.5), eluted with a linear gradient to 1 M NaCl. Fractions containing purified peptide were pooled and dialyzed against 20 mM Mops, 100 mM KCl, 1 mM TCEP, 1 mM NaN3 (pH 7.2). To prepare the tag-less CARMIL1 peptide, the 6x His tag was cleaved from the peptide with GST-tagged PreScission protease (Cytiva). The GST-tagged PreScission protease was removed using Glutathione Superflow Agarose (Pierce Chemical Company). The peptides were stored at −70 °C. Molar mass of the purified peptides was confirmed by MALDI mass spectrometry.

#### AlexaFluor-594 labeling of CBR126

Purified preparations of tag-less CBR126 WT and CBR126 mut were dialyzed against 20 mM sodium phosphate, 0.15 M NaCl, 1 mM NaN3 (pH 7.4). Six to ten mol of Alexafluor 594 C5 maleimide (Thermo Fisher Scientific) was added per mol of CBR, and the solution was incubated overnight at 4 °C. The labeled CBRs were dialyzed against 20 mM sodium phosphate, 0.15 M NaCl, 1 mM NaN3, 10 mM DTT (pH 7.4), then 20 mM Mops, 100 mM KCl, 1 mM TCEP, 1 mM NaN3 (pH 7.2), and stored at −70 °C.

#### His-tagged VVCA

His-tagged VVCA (pBJ 2534) was composed of N-terminal GST-LEVLFQGP (PreScission protease cleavage site)-HHHHHH-human N-WASP (UniProt O00401) P392 to D505. Codon-optimized DNA was synthesized and inserted into a pRSF-1b vector (Novagen) by Azenta Life Sciences. The recombinant protein was expressed in *E. coli* NiCo21 (DE3) (New England Biolabs). The cells were lysed and the soluble fraction of the lysate was purified with Glutathione Sepharose 4 Fast Flow (Cytiva) in 20 mM sodium phosphate, 0.1 M NaCl, 0.1 mM EDTA, 1 mM NaN3, 10 mM DTT (pH 7.8), and eluted with 20 mM sodium phosphate, 0.1 M NaCl, 0.1 mM EDTA, 1 mM NaN3, 10 mM DTT, 10 mM reduced glutathione (pH 7.8). The protein was then purified by anion exchange chromatography using a POROS GoPure 50 HQ column (Applied Biosystems) in 20 mM Tris–HCl, 1 mM NaN3, 10 mM DTT (pH 7.5), eluted with a linear gradient to 1 M NaCl. Fractions containing purified peptide were pooled, dialyzed against 20 mM Mops, 100 mM KCl, 1 mM TCEP, 1 mM NaN3 (pH 7.2), and stored at −70 °C.

### Actin polymerization assays

Pyrene-labeled F-actin seeds were prepared by adding 1 mM MgCl2, 1 mM EGTA, 50 mM KCl, and 5 μM phalloidin to 5 μM G-actin (5% pyrene label) and incubating at room temperature overnight. To measure elongation rates, CP (0 or 5 nM) and tag-less CARMIL1 fragments (0–2500 nM), with or without 1 mg/ml 50% DOPC, 50% DOPS liposomes were then added to 2.4 μM G-actin (5% pyrene label). To initiate polymerization, pyrene-labeled F-actin seeds were added at a concentration of 1.25 μM. Elongation rates were measured at 25 °C using time-based scans on a steady state spectrofluorometer with excitation at 368 nm and emission detected at 386 nm (PTI QuantaMaster 8000 with Felix GX software).

### Isothermal calorimetry

ITC experiments were performed on a MicroCal MicroCalorimeter (Malvern Panalytical). CP (2 μM) was titrated with tag-less CARMIL1 CBR126 WT or tag-less CBR126 mut at 25 °C in 20 mM Mops, 300 mM KCl, 1 mM TCEP, 1 mM NaN3, 0.005% Tween20 (pH 7.2). Binding constants were determined by fitting the change in enthalpy to a single site binding model using MicroCal ITC Origin analysis software.

### Liposome preparation

#### Liposomes for actin polymerization experiments

Hundred milligrams of 18:1 (Δ9-Cis) PC (DOPC) [1,2-dioleoyl-sn-glycero-3-phosphocholine in chloroform (Avanti Research 850375C Alabaster)] and hundred milligrams of 18:1 PS (DOPS) [1,2-dioleoyl-sn-glycero-3-phospho-L-serine (sodium salt) in chloroform (Avanti Research 840035C)] were combined and dried with Argon gas followed by further drying under vacuum. Two milliliters of 20 mM Mops, 300 mM KCl, 1 mM TCEP, 1 mM NaN3 (pH 7.2) was added to the dry lipids. The lipids were resuspended by vortexing and sonication. The lipid suspension was then frozen in a dry ice ethanol bath and thawed at room temperature. The freeze-thaw cycle was repeated four times. The lipid suspension was then extruded seven times through a 0.2 μm polycarbonate membrane (Avanti Research 610006). 1.8 ml of the liposome suspension was added to 7.2 ml 20 mM Mops, 300 mM KCl, 1 mM TCEP, 1 mM NaN3 (pH 7.2) and was stored at −70 °C. Prior to use in assays, the liposome suspension was thawed and the extrusion was repeated.

#### Liposomes for bead and sedimentation experiments

Lipids were mixed together to give a final total concentration of 5 mM at the following ratios: 44% 1,2-dioleoyl-sn-glycero-3-phosphocholine (18:1 (Δ9-Cis) PC, DOPC, Avanti Research, #850375), 5% 1,2-dioleoyl-sn-glycero-3-[(N-(5-amino-1-carboxypentyl)iminodiacetic acid)succinyl] (nickel salt) (18:1 DGS-NTA(Ni), Avanti Research, #790404), 50% 1,2-dioleoyl-sn-glycero-3-phospho-L-serine (sodium salt) (18:1 PS, DOPS, Avanti Research, #840035), 1% 1,2-dioleoyl-sn-glycero-3-phosphocholine-N-(Cyanine 5) (18:1 Cyanine 5 PC, Avanti Research, #850483), or 1% 1,2-dioleoyl-sn-glycero-3-phosphoethanolamine-N-(1-pyrenesulfonyl) (ammonium salt) (18:1 pyrene PE, Avanti Research, #810331). The lipid mixture was dried under argon and then under vacuum for 1 h. Lipids were resuspended in Mops buffer (20.0 mM Mops, 100 mM KCl, 1.0 mM NaN_3_, 1.0 mM TCEP, pH 7.2) and cycled through rounds of vortexing for 30 s and sonication for 5 min three times. Liposomes underwent five freeze/thaws using an ethanol/dry ice bath. Liposomes were then passed through a hand-extruder (Genizer, Avanti Research, #610000) and through a 0.2 μm polycarbonate membrane (Avanti Research, #610006) nine times and collected for use. Liposome preparation for sedimentation assays was the same except the lipids were 50% DOPC and 50% DOPS at a total final concentration of 20 mg/ml.

### Liposome sedimentation assays

Purified proteins at 2 μM or as labeled in the figures were mixed together with and without 10 mg/ml liposomes (preparation described above) at room temperature for 15 min. Samples were then centrifuged at 53,000 rpm for 30 min at 4 °C using a tabletop ultracentrifuge and fixed angle rotor. A percentage, 35%, of the supernatant was collected for SDS-PAGE gel analysis. The rest of the supernatant was discarded, and the remaining pellet was resuspended for SDS-PAGE gel analysis. Samples and protein standards were run on 15% gels and stained with Blazin’ Blue Protein Gel Stain (Goldbio #P-810-1). Gels were scanned and gel bands were analyzed using the Gel Analysis Tools in ImageJ. The percentage of protein that pelleted was calculated by comparing to a total protein standard loaded on the same gel.

### Protein-coating of beads

Silica beads with a diameter of 2.5 μm (Bangs Laboratory, SS05000) were sonicated for 10 min, centrifuged (3 min, 5000 rpm), and washed using Mops buffer (20.0 mM Mops, 100 mM KCl, 1.0 mM NaN_3_, 1.0 mM TCEP, pH 7.2). Beads were coated with poly-L-lysine (0.01% solution, Sigma, P4707) by incubation for 1 h at room temperature (r.t.). Poly-L-lysine–coated beads were then coated with lipid by mixing 3.5 μl of beads (8.0 × 10^6^ beads/μl, 6.2 × 10^6^ beads/μl final concentration) with 5 μl of 5 mM liposomes (preparation described above) in 45 μl Mops buffer, sonicating three times for 10 s, and incubating for 1 h at r.t. with frequent gentle agitation. Lipid-coated beads were washed twice in Mops buffer. Beads were suspended in Mops buffer containing the following concentrations of 6xHis-tagged fusion proteins: 2 μM His-VVCA, 0 to 2 μM His-CBR126, 0 to 2 μM Hexa His tag peptide (APExBIO, A6006). Hexa His tag peptide was used as a placeholder when the concentration of His-CBR126 was less than 2 μM, so that the total concentration of 6xHis was 4 μM in all samples during the coating process. Beads were incubated with 6xHis fusion proteins for 2 h on ice, with frequent agitation to maintain the beads in suspension. Beads were centrifuged and washed once in assay buffer (no ATP) with 1% BSA, followed by a second wash step in assay buffer (no ATP) with 0.1% BSA. Protein-coated beads were then suspended in assay buffer with 0.1% BSA and 1 mM ATP. Protein-coated beads were used within 1 or 2 days.

For assays that utilized tag-less CBR126, beads were first precoated with His-VVCA and Hexa His and washed as previously described. Next, these beads were incubated with various concentrations of tag-less fluorescently labeled CBR126 wt or CBR126 mut for 15 min at r.t., followed by a centrifugation step and resuspension in assay buffer with 0.1% BSA and 1 mM ATP. We confirmed that CBR126 wt stayed associated with lipid beads over the course of several hours, but beads were always used immediately for the assays reported.

### Bead-based actin polymerization microscopy assays

For bead-based actin polymerization assays, protein-coated beads (∼3.2 × 10^5^ beads or ∼1.6 × 10^4^ beads/μl) were mixed with 100 nM Arp2/3 complex, CP (0–200 nM), 5 μM profilin, 5 μM actin (10% Alexa 488-labeled actin), and V-1 (0–5000 nM) in assay buffer containing 0.2% methylcellulose (cP 400), 2.5 mg/ml BSA, and 20 mM β-mercaptoethanol. To ensure that relevant complexes formed before the start of the actin polymerization reaction, we premixed three pairs of components: Arp2/3 complex with VVCA-coated beads, actin monomers with profilin, and CP with V-1. The pairs were incubated for 15 min at RT. All three of the premixes were then combined into one vessel to start the polymerization reaction. After 15 min at RT, the reaction mixture was mounted between a coverslip and a glass slide, sealed using clear fingernail polish and imaged after another 15 min. Alternatively, the reaction was arrested after 30 min of incubation by adding 10 μM phalloidin and 10 μM Latrunculin B, mounted between a coverslip and a glass slide, and imaged immediately. These reaction times were chosen after pilot experiments testing a range of times.

Samples were imaged using a 60× 1.45 NA oil objective on an inverted Olympus IX81 microscope using a mercury lamp for excitation. Images were collected in wide-field fluorescence and bright-field modes with a Hamamatsu EM-CCD (C9100) camera using Micro-Manager software (39). Digital images were analyzed using Fiji software (40).

### Analysis of fluorescence area, circularity, and intensity around beads

Two areas of actin fluorescence were determined, the area of brightest fluorescence near the bead surface and the total area including the diffuse cloud of actin. For the area of brightest fluorescence near the bead surface, the area was outlined using the freehand drawing tool and the Measure tool in Fiji (40). When beads were surrounded by diffuse clouds of actin fluorescence, the edge of the actin cloud was less clear, so a threshold level was set and used as a mask to demarcate the edge. The freehand drawing tool was used to outline around the threshold mask to determine the area of the actin cloud.

To determine the shape of the fluorescence (circularity) for the area of brightest fluorescence closest to the beads, the Freehand tool was used to draw around the area, followed by the Measure tool to determine the circularity of each shape. Circularity is defined by ImageJ as *circularity = 4pi(area/perimeterˆ2)*, with the value of 1 indicating a perfect circle.

To measure the total actin fluorescence associated with each bead, a 40 × 40 μm box was drawn centered around the bead, and the value of the integrated density was recorded. Next, an ∼50 × 50 μm box was drawn around the outside of an ∼40 × 40 μm box to determine the background value surrounding each bead. The background was calculated using the following equation:

Fbackground = (Fo - Fi)∗(1615 μm^2^/(2512 μm^2^ - 1615 μm^2^)) Note: 1615 and 2512 correspond to the areas of the smaller and larger boxes, respectively.

In this equation, Fo is the outer box fluorescence and Fi is the inner box fluorescence. The total fluorescence of the bead was then calculated as:

Ftot = Fi – Fbackground

### Scanning electron microscopy of lipid-coated bead surfaces

Treated 2-μm silica bead samples (hereafter referred to as samples) were delivered in 217 mOsm/L Mops buffer and fixed in 0.1% osmium tetroxide for 30 min in Hanks Buffered Saline Solution adjusted to 200 mOsm/L. The samples were rinsed three times in the adjusted Hanks Buffered Saline Solution for 10 min each, then affixed to poly-L-lysine (1 mg/ml)–coated cover slips. The samples were dehydrated through a graded ethanol (ETOH) series (30%, 50%, 70%, 90%, and 100%) for 10 min at each concentration, repeated four times. The final two dehydrations in 100% ETOH were performed with ETOH dried over 3 Å molecular sieves (Sigma-Aldrich). Once fully dehydrated, the samples were placed in a Leica EM CPD 300 critical point dryer set to perform 12 CO_2_ exchanges at the slowest speed. The sample coverslips were mounted on aluminum pin mounts with colloidal silver paint (Electron Microscopy Sciences) and then coated with 10 nm of carbon and 6 nm of iridium using a Leica ACE 600 vacuum coater. Scanning electron microscopy images were captured using a Merlin (Carl Zeiss) field emission-scanning electron microscopy at a resolution of 2 nm at 3 kV and 200 pA probe current with a secondary electron detector.

## Data availability

The data used and analyzed during the current study are available from the corresponding author on reasonable request.

## Supporting information

This article contains supporting information (https://www.sample-size.ent/).

## Conflict of interests

The authors declare that they have no conflict of interests with the contents of this article.
