# Supplementary material for: CARMIL membrane-binding domain regulates capping protein and actin assembly
Source: J Biol Chem. 2026 Apr 22;302(6):111484. doi: 10.1016/j.jbc.2026.111484 (PMC13208823; doi:10.1016/j.jbc.2026.111484)
Supplement: Supplementary Figures and Tables [file mmc1.pdf]

Supplementary Information

Suppl. Table 1. Beads with asymmetric actin tail formation / total number beads counted for three separate experiments. His-tagged CBR126 wt compared with His-tagged CBR126 mut. Data for each experiment are listed in separate rows, followed by totals for the three experiments. Percentages are listed in the next row, and 95% confidence limits are in the final row, calculated from a web site cited as: Kohn MA, Senyak J. Sample Size Calculators [website]. UCSF CTSI. 16 February 2026. Available at https://www.sample-size.net/ [Accessed 31 March 2026].

Experiment #	CP only	CP + V-1	CP + V-1 + CBR126 wt	CP + V-1 + CBR126 mut
92425	25/25	0/10	10/18	14/19
30526	22/22	0/20	16/21	11/20
31126	53/53	1/21	21/24	17/30
Totals	100/100	1/51	47/63	42/69
Percentage	100	2	75	61
95% Conf Interval	96 - 100 *	0 –10	62 – 85	48 – 72

* One-sided 97.5% confidence interval.

Suppl. Table 2. Beads with asymmetric actin tail formation / total number beads counted for three separate experiments. Untagged CBR126 wt compared with untagged CBR126 mut. Data for each experiment are listed in separate rows, followed by totals for the three experiments. Percentages are listed in the next row, and 95% confidence limits are in the final row, calculated from a web site cited as: Kohn MA, Senyak J. Sample Size Calculators [website]. UCSF CTSI. 16 February 2026. Available at https://www.sample-size.net/ [Accessed 31 March 2026].

Experiment #	CP only	CP + V-1	CP + V-1 + CBR126 wt	CP + V-1 + CBR126 mut
112025	13/13	0/13	13/31	3/22
30526	22/22	0/20	20/21	5/23
31126	53/53	1/21	39/40	10/27
Totals	88/88	1/54	72/92	18/72
Percentage	100	2	78	25
95% Conf Interval	96 – 100 *	0 – 10	68 - 86	16 - 37

* One-sided 97.5% confidence interval.

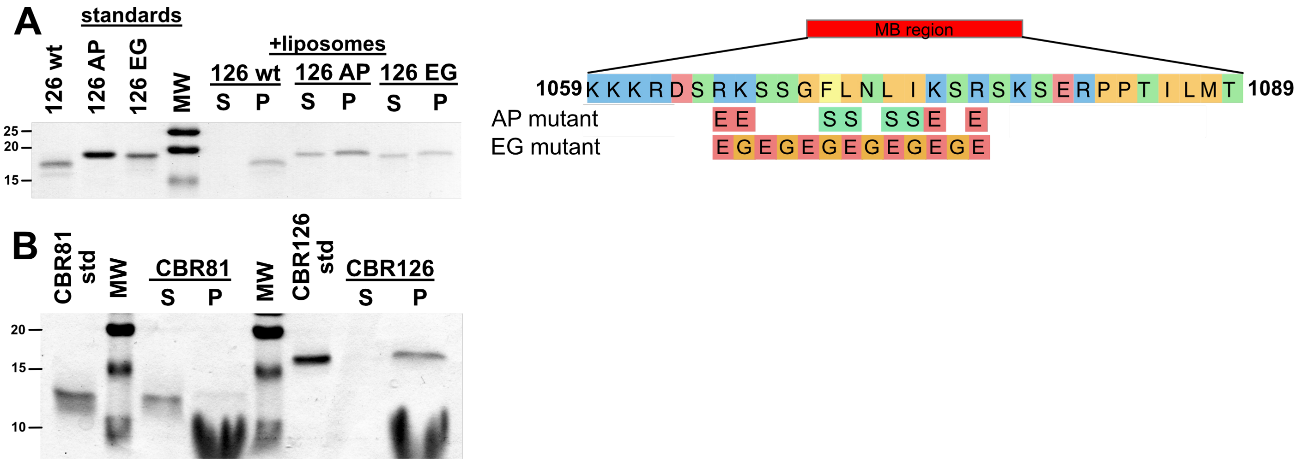

Suppl. Figure 1. MB domain mutants and membrane binding. A. SDS-PAGE of sedimentation assays of MB mutants with two different sets of changes to a core 13-aa sequence, diagrammed on the right. One set, labeled “AP mutant”, reverses basic/hydrophobic residues to acidic and polar residues, and the other set, labeled “EG mutant”, consists of wholesale changes of residues to an alternating pattern of E and G. Each mutant shows decreased but not abrogated binding to liposomes in the sedimentation assay. **B.** SDS-PAGE of sedimentation assay showing that CBR81, which lacks the MB domain, did not associate with liposomes.

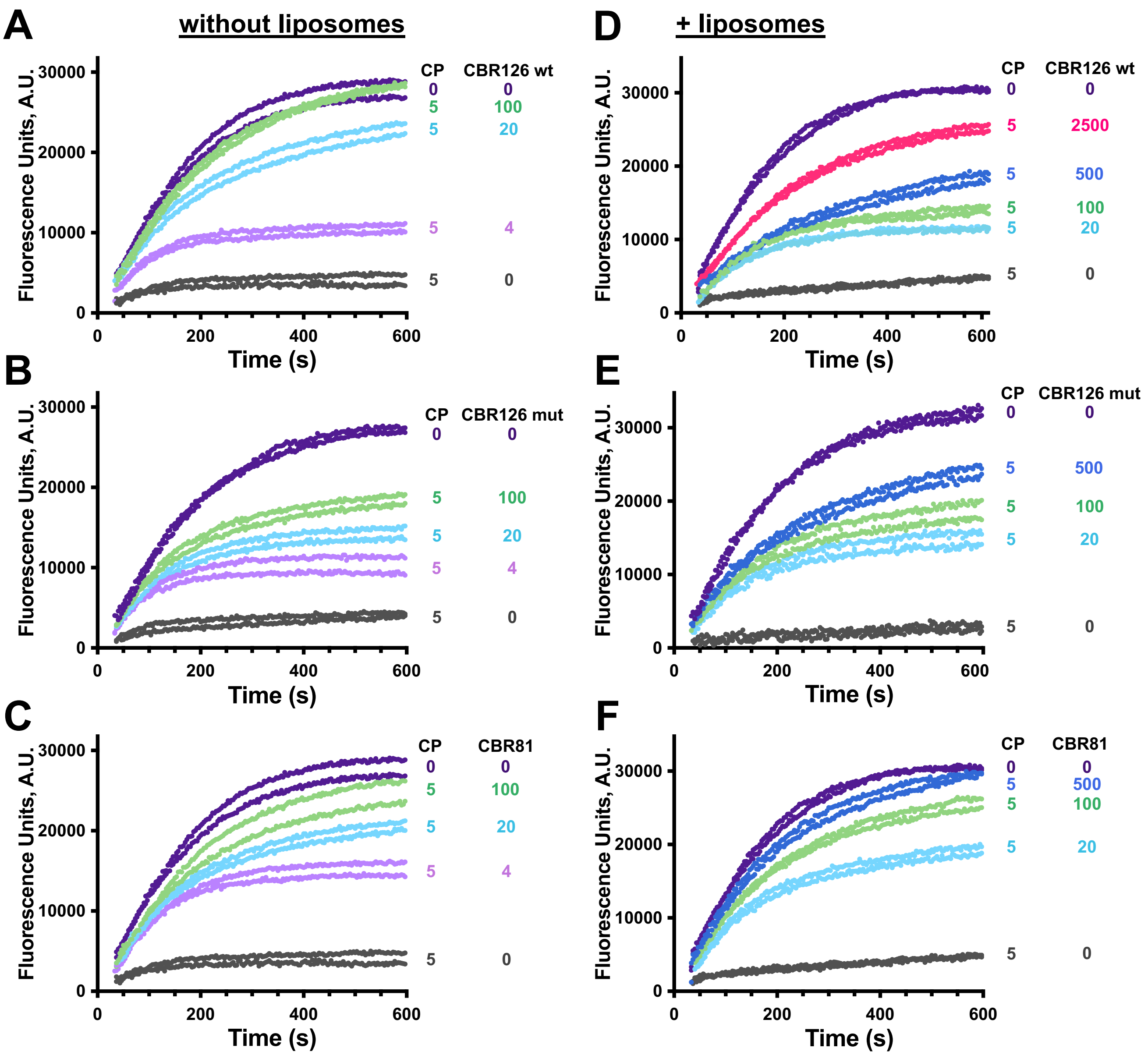

Suppl. Figure 2. Actin polymerization capping assays – full set of CBR titration curves. Increasing concentrations (nM, as indicated) of CBR126 wt (wild type) (**A**), CBR126 mut (mutant) (**B**), and CBR81 (**C**) were added to CP to inhibit its barbed-end actin capping activity. **D-F.** The same experiment as panels **A-C**, except in the presence of liposomes. Pyrene actin fluorescence (arbitrary units) is plotted vs time (s).

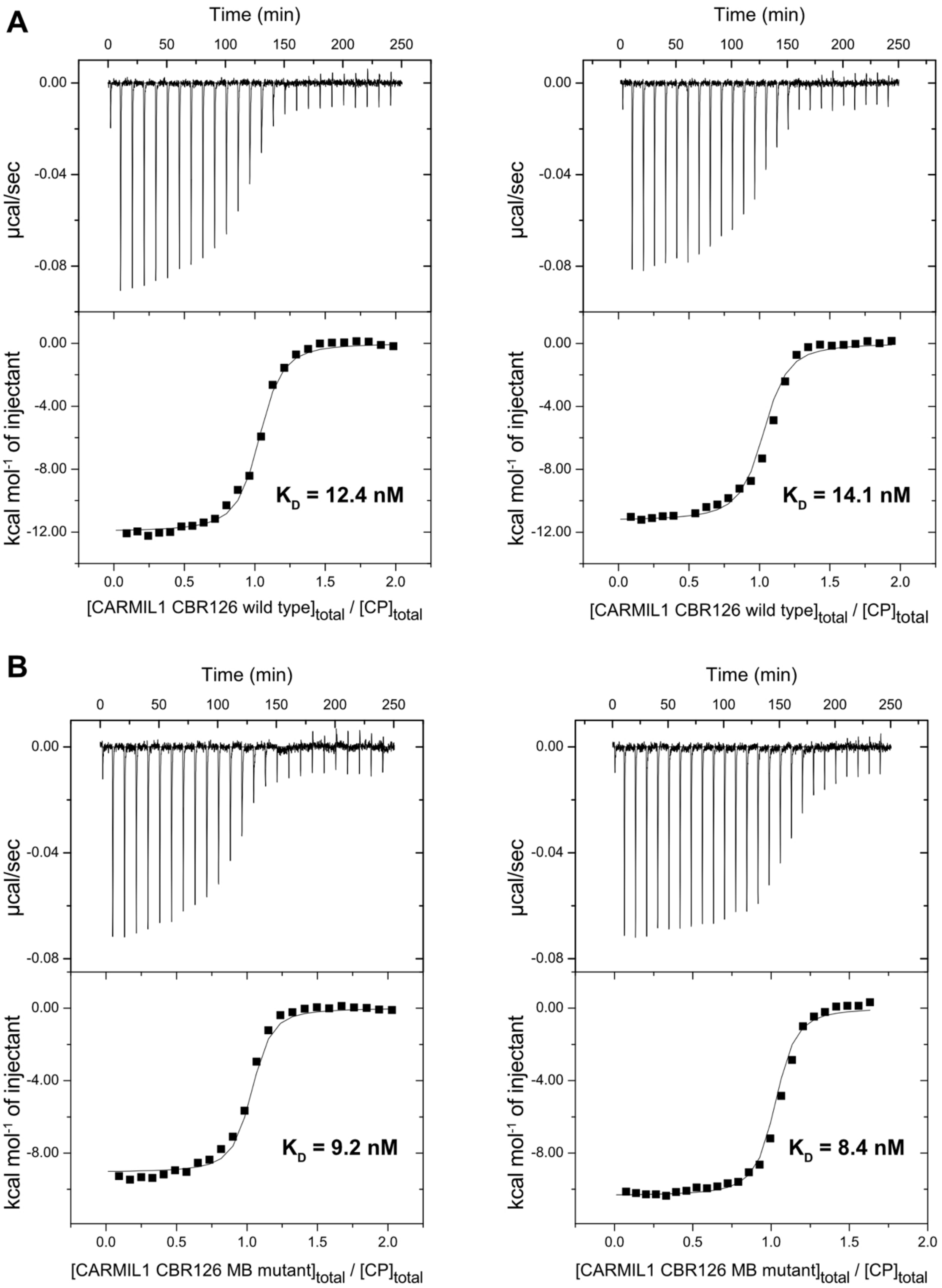

Suppl. Figure 3. Binding affinities of CP for CBR126 wild type and CBR126 mut are similar. ITC traces of CP titrated with CBR126 wild type (panel A) or CBR126 mut (panel B). Upper panels are raw traces of thermograms, with differential power for each injection of CBR. Lower panels are binding isotherms, with integrated enthalpy vs molar ratio of reactants. Fitted values for K_D, with a stoichiometry of 1.0 are listed. Left and right are independent replicates.

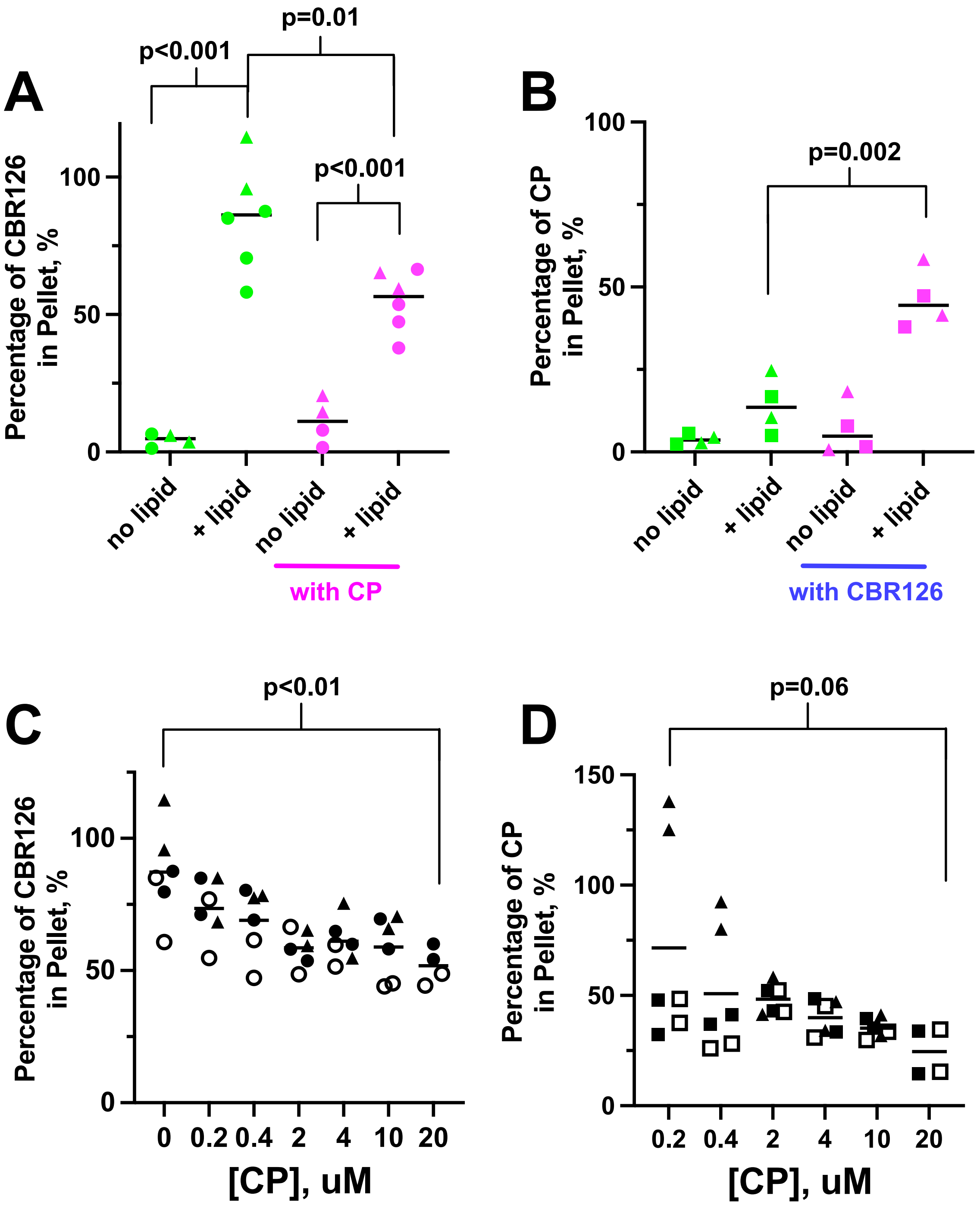

Suppl. Figure 4. CP and liposomes appear to compete for CBR126 binding. For all panels the ordinate is the fractions of CBR126 or CP that pellets with liposomes, plotted as a percentage of the total. The horizontal black bar is the median, and p values are calculated from a Welch's t test analysis. **Panel A.** CBR126 alone, without CP, sediments with liposomes when lipids are present, but not in the absence of lipids (green points). When CP is added to the reaction mixture, the amount of CBR126 that pellets with the liposomes is less (pink points). **Panel B.** CP pellets with liposomes when CBR126 is present (pink points) but not in its absence (green points). For panels A and B, independent experiments are plotted as separate points, and triangles represent a set of technical replicates. **Panel C.** Fraction of CBR126 that pellets decreases as a function of increasing concentrations of CP. **Panel D.** Fraction of CP pelleting with CBR126 decreases with increasing concentrations of CP. The total amount of CBR126 was constant. For panels C and D, experiments were performed in triplicate, and technical replicates are shown as different shapes.

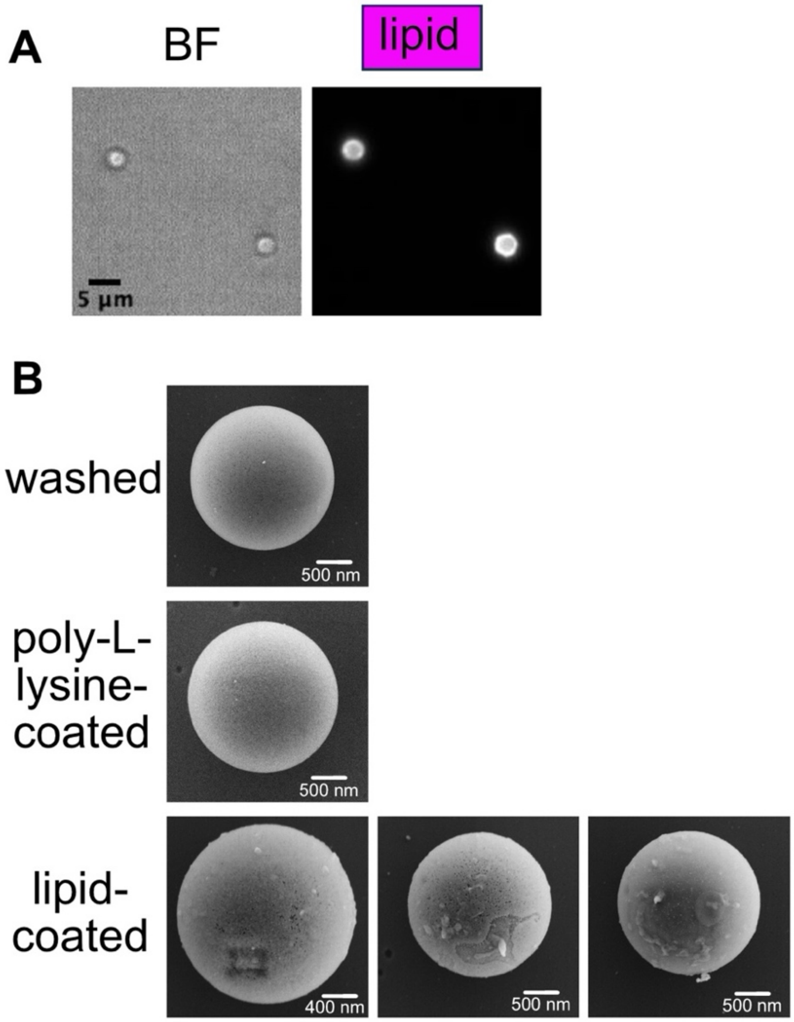

Suppl. Figure 5. Images of lipid-coated silica beads. A. Brightfield (BF) and fluorescence image of beads coated with a lipid mixture containing 1% Cy5-PC. **B.** Scanning electron micrograph (SEM) images of silica beads that are not coated (washed), poly-L-lysine-coated, or lipid-coated.

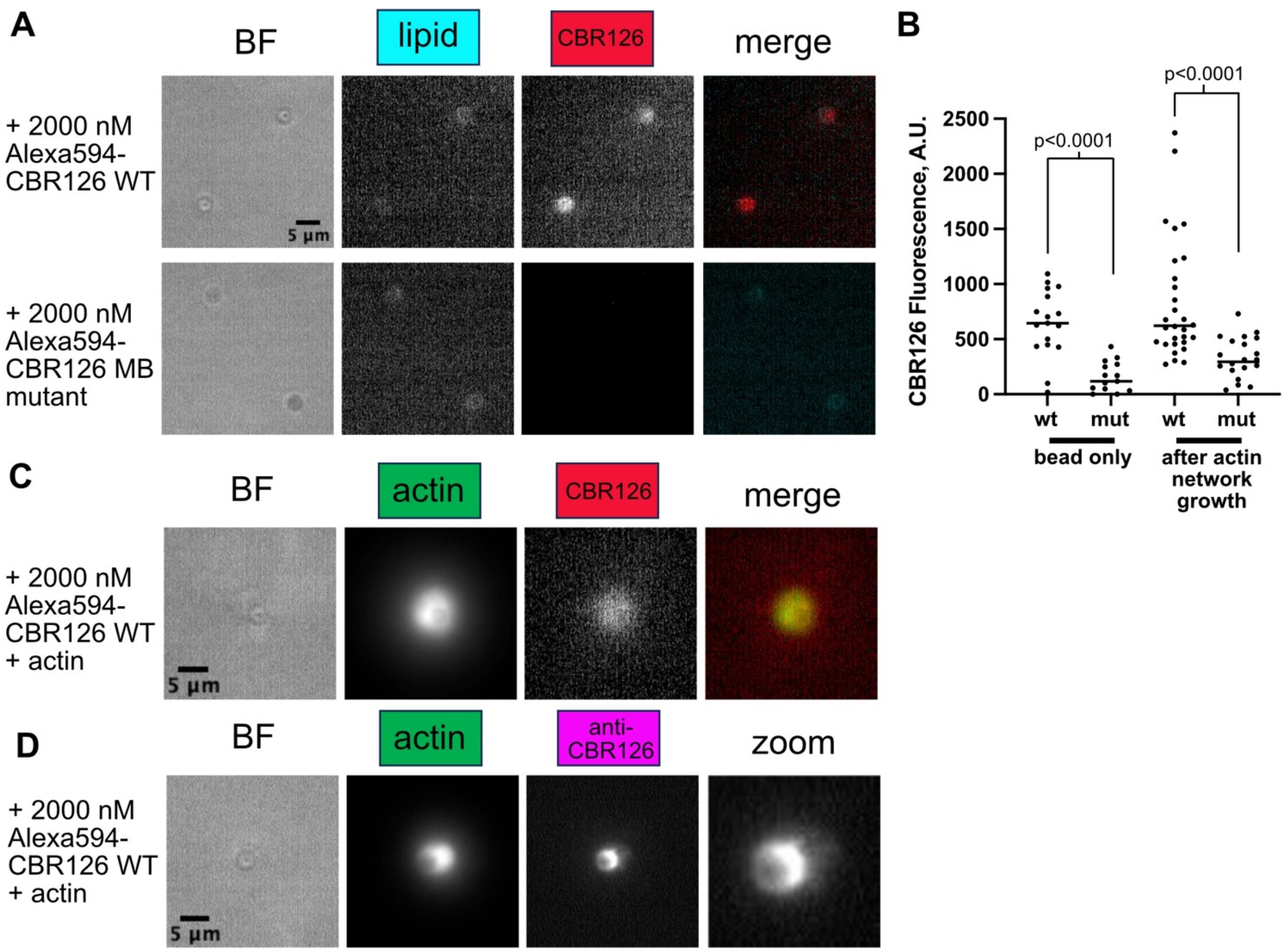

Suppl. Figure 6. Localization of fluorescent CBR126 without His tag before and after actin polymerization. A. CBR126 wt localizes to the surface of the lipid-coated bead before addition of actin. CBR126 mut shows no localization. B. Fluorescence of Alexa-594-CBR126 was measured for lipid beads after 15 min. incubation and wash step. CBR126 wt is recruited to the beads but CBR126 mut is not. These beads were then used in actin network growth assays. **C.** After actin polymerizes, CBR126 wt localization is lost at the bead surface, at times co-localizing with the F-actin. **D.** Localization of CBR126 by antibody staining of CBR126 confirms colocalization with actin. Image labeled “zoom” is a higher magnification of the anti-CBR126 image.
